# Tetrahedral amorphous carbon prepared filter cathodic vacuum arc for hole transport layers in perovskite solar cells and quantum dots LEDs

**DOI:** 10.1080/14686996.2019.1694841

**Published:** 2019-11-22

**Authors:** Hae-Jun Seok, Yong-Jin Kang, Jongkuk Kim, Do-Hyeong Kim, Su Been Heo, Seong Jun Kang, Han-Ki Kim

**Affiliations:** a School of Advanced Materials Science and Engineering, Sungkyunkwan University, Suwon-si, Republic of Korea; b Surface Engineering Department, Implementation Research Division, Korea Institute of Materials Science (KIMS), Changwon-Si, Republic of Korea; c Energy & New Industry Laboratory, Korea Electric Power Research Institute, Daejeon, Republic of Korea; d Department of Advanced Materials Engineering for Information and Electronics, Kyung-Hee University, Yongin-si, Republic of Korea

**Keywords:** Tetrahedral amorphous carbon (ta-C) film, hole transport layer (HTL), hole injection layer (HIL), perovskite solar cells, quantum dot LEDs, 104 Carbon and related materials

## Abstract

(ta-C) films coated through the filtered cathodic vacuum arc (FCVA) process as a hole transport layer (HTL) for perovskite solar cells (PSCs) and quantum dot light-emitting diodes (QDLEDs). The p-type ta-C film has several remarkable features, including ease of fabrication without the need for thermal annealing, reasonable electrical conductivity, optical transmittance, and a high work function. X-ray photoelectron spectroscopy and ultraviolet photoelectron spectroscopy examinations show that the electrical properties (sp^3^/sp^2^ hybridized bond) and work function of the ta-C HTL are appropriate for PSCs and QDLEDs. In addition, in order to correlate the performance of the devices, the optical, surface morphological, and structural properties of the FCVA-grown ta-C films with different thicknesses (5 ~ 20 nm) deposited on the ITO anode are investigated in detail. The optimized ta-C film with a thickness of 5 nm deposited on the ITO anode had a sheet resistance of 10.33 Ω^−2^, a resistivity of 1.34 × 10^−4^ Ω cm, and an optical transmittance of 88.97%. Compared to the reference PSC with p-NiO HTL, the PSC with 5 nm thick ta-C HTL yielded a higher power conversion efficiency (PCE, 10.53%) due to its improved fill factor. Further, the performance of QDLEDs with 5 nm thick ta-C hole injection layers (HIL) showed better than the performance of QDLEDs with different ta-C thicknesses. It is concluded that ta-C films have the potential to serve as HTL and HIL in next-generation PSCs and QDLEDs.

## Graphical abstract

**Figure:**
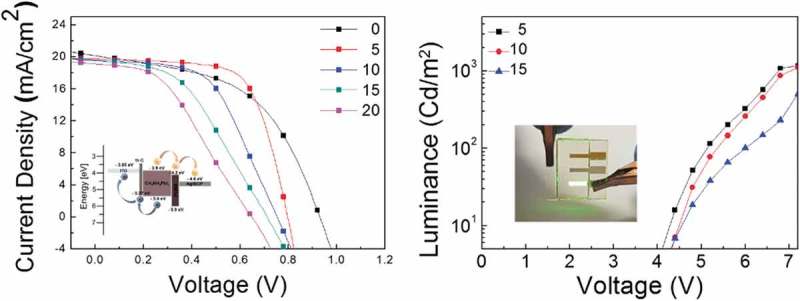

## 1. Introduction

In recent years, organic-inorganic metal halide perovskite solar cells (PSCs) and quantum dot light emission diodes (QDLEDs) have attracted substantial research interest for use in next-generation photovoltaics and flat panel displays because of the high solar-to-electric power conversion efficiency (PCE) of PSCs and interesting property characteristic, such as good color purity with narrow full-width at half maximum (FWHM) emission of QDLEDs [1–13]. In order to capture the main market of Si-based solar cells, it is necessary to fabricate cost-effective and high efficient photovoltaics that perform comparably to Si-based solar cells. As one of the most promising photovoltaics, PSCs have been studied extensively, due to their large absorption coefficient, carrier mobility, long carrier diffusion lengths, and low nonradiative recombination rate [14–18]. The power conversion efficiency of this type of photovoltaic device has been improved from an initial efficiency of 3.8% up to a certified efficiency of 25.2% [19–26]. In addition, QDLEDs have attracted increasing attention as an alternative to organic light-emitting diodes. In particular, semiconductor quantum dots (QDs) have been the subject of many intensive studies in recent years, due to their unique optical properties, such as size-dependent emission wavelength, narrow emission spectrum, high luminescent efficiency, and good stability [27–29].

Researchers have achieved great improvements in the performances of both PSCs and QDLEDs as next-generation devices by improving the active layer materials, adjusting the device architectures, optimizing the carrier transport layer materials (CTLs), promoting and balancing the carrier injection efficiency, and so on. To date, high-efficiency PSCs and QDLEDs have generally adopted organic-inorganic composite multilayer structures, in which the CTLs consist of organic polymers and inorganic metal oxide – as a hole transport layer (HTL) in the case of PSCs or as a hole injection layer (HIL) in the case of QDLEDs – exhibiting outstanding device performance [30–37]. In particular, the important roles of the HTL include not only collecting the holes but also blocking the electrons, i.e., hole collection in PSCs and hole injection for QDLEDs. They also prevent the active layer from directly contacting the electrodes, thereby reducing the recombination of photocarriers. Moreover, the electrical and optical properties of the HTL can have major impacts on the performances of PSCs and QDLEDs. Thus, selecting an HTL or HIL with controlled physical properties is essential to understanding the operation of devices in terms of carrier separation, transport, extraction, injection, and recombination. However, the limited hole transport or injection efficiency is one of the large obstacles to further improvements in the performances of PSCs and QDLEDs. For instance, the inherent acidity of PEDOT:PSS can damage indium tin oxide (ITO) anodes by dissolving indium species, and the hygroscopic nature also accelerates the diffusion of indium, which both significantly affect the device stability [38–40]. In attempts to address these problems, inorganic metal oxides (NiO, MoO_3_, WO, and V_2_O_5_) have been successfully adopted in PSCs and QDLEDs due to their suitable work function, chemical stability, and good carrier transfer capability [41–47]. However, solution-based metal-oxide HTL has critical problems with large area coating and ensuring exact control of the thickness and composition. As another replacement of PEDOT:PSS, carbon-based materials (tetrahedral amorphous carbon, graphene, fullerene, and carbon nanotubes) have also been investigated. In particular, among the other carbon-based alternative materials, a tetrahedral amorphous carbon (ta-C) film prepared using the filter cathodic arc vacuum (FCVA) process at room temperature is an interesting alternative hole transport and injection layer for solar cells and flat panel displays. To date, ta-C films have mainly been used as a very effective protective and hard coating in many applications due to their outstanding hardness that is similar to that of a diamond. According to the results of recent studies, ta-C films have high sp^3^ bonding content (40% or more) with hardness that increases with sp^3^ bonding content [48–50]. In addition, ta-C also contains a small fraction of sp^2^ bonding. It has been shown that the sp^3^ fraction controls the mechanical properties of ta-C, while sp^2^ sites primarily control the optical and electrical properties [51–54]. These sp^3^ and sp^2^ fractions in ta-C films result in mechanical, optical, and electrical properties that range from a hardness of ~70 GPa and Young’s modulus of ~700 GPa, chemical inertness, optical band gap of 2.0 eV, and electrical resistivity of ~10^9^ Ω cm. These unique electrical, optical, mechanical, structural, and morphological properties have led to considerable interest in ta-C films [55–59]. In general, undoped ta-C has been shown to be intrinsically p-type [60]. The electronic properties of ta-C can also be altered by a doping process using V and III group elements. N-type doping of ta-C has been observed when nitrogen is incorporated into the ta-C films [61]. P-type ta-C films using boron incorporation continue to be an area of great interest and relevance to establishing ta-C as a viable material for electronic applications [60,61].

In this study, the feasibility of using carbon-based material ta-C prepared through the FCVA process as the hole transport or injection layer in PSCs and QDLEDs was investigated. We show the electrical, optical, and surface morphological properties of ta-C thin films, as a function of the ta-C thickness to determine the optimum thickness. In addition, X-ray photoelectron spectroscopy (XPS; K-Alpha, Thermo Fisher Scientific, USA) analysis showed the correlation between HTL properties and sp^3^/sp^2^ hybridized bond ratio in ta-C films. With an optimized thickness, the ta-C was applied in a PSC and QDLED in order to investigate its influence on device performance. Furthermore, we comprehensively studied the microstructure of ta-C films and interfaces in PSCs using transmission electron microscopy (TEM) and ultraviolet photoelectron spectroscopy (UPS; ESCALB-250Xi, Thermo Fisher Scientific, USA) to elucidate the hole extraction and injection mechanisms. Based on the performances of PSCs and QDLEDs with ta-C HTL, we propose ta-C film as a promising HTL to substitute PEDOT:PSS in PSCs and QDLEDs.

## 2. Experimental details

### 2.1. Fabrication of ITO electrodes on glass substrates by magnetron sputtering

**Figure 1.**
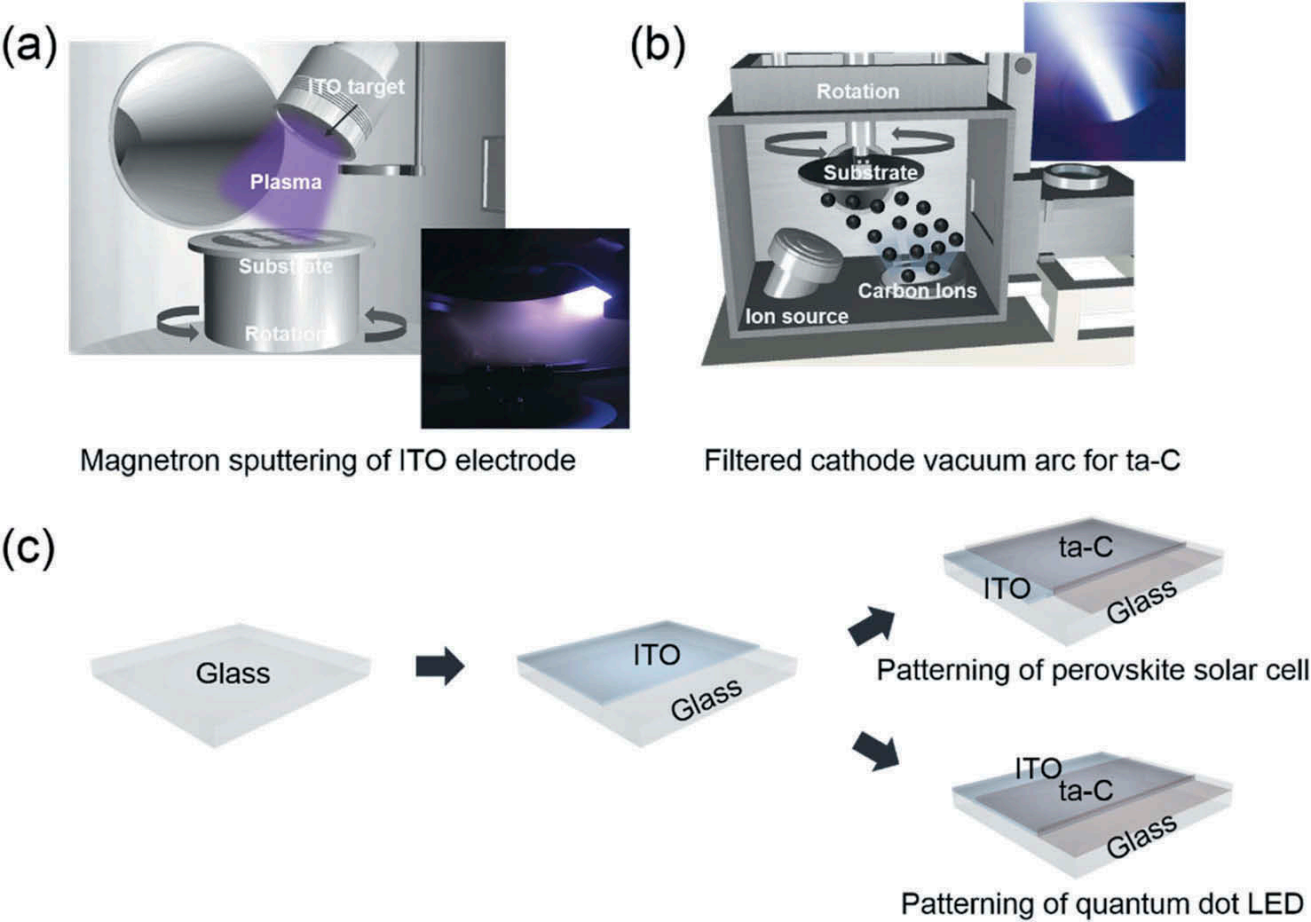
(a) Schematic of the DC magnetron sputtering system employed for deposition of the ITO anode on glass substrates at room temperature. Schematic of the ITO sputtering plasma FCVA process for coating of ta-C layers with different thicknesses on the ITO anode. The inset shows the arc plasma beam. (c) Schematic fabrication process of the patterned ta-C/ITO anode for PSC and QDLED devices.

First, the glass substrates were cleaned using standard cleaning procedures (acetone-isopropyl alcohol-acetone-methanol) with ultrasonic cleaner for 10 min. Then, the glass was rinsed with deionized water. After loading the cleaned glass substrates from the load lock chamber, the bottom ITO anode was deposited on the glass substrate by applying a constant DC power of 100 W, an Ar flow rate of 20 sccm, and a working pressure of 3 mTorr using a 4-inch ITO target (Dasom RMS) at room temperature. During sputtering, constantly rotating the glass substrate at 25 rpm improves the film uniformity with 25°-tilted cathode guns, as shown in Figure 1(a). After sputtering, the ITO anodes were annealed at a temperature of 600°C for 10 min to improve their electrical and optical properties.

### 2.2. Coating of ta-C layer by single-cathode FCVA

Figure 1(b) shows the schematic fabrication process that directly deposits the ta-C layer using a single-cathode of FCVA system on the sputtered ITO anode. The vacuum arc source is used with a system to separate charged carbon ions from neutral atoms and macro- or micro-particles. A carbon target with a diameter of 55 mm and of 99.99% purity is mounted on the bottom right side of the chamber. Those macro-particles and neutral carbon could be filtered out by a T-shaped filter. Therefore, only carbon with controlled energy range ions will be deposited on the ITO anode. The magnetic coil current is fixed to 5 A. The substrate holder was placed horizontally on a sample carrier for high-speed coating at a working pressure of 1 × 10^−5^ Torr without the need for intentional substrate heating. During the deposition, a duct bias of 20 V and a substrate bias of 500 V were applied. The Ar gas flowed at a rate of 2 sccm for stable arc plasma generation. The thickness of ta-C was controlled by varying the deposition time between 30 and 120 s. To apply the ta-C coated ITO anode as a hole transport or injection layer for PSCs and QDLEDs, we fabricated patterned ta-C/ITO anodes with a size of 1.5 × 1.5 mm^2^, as shown in Figure 1(c).

### 2.3. Analysis of single ta-C layers and ta-C/ITO anodes

XPS was employed to probe the sp^3^/sp^2^ hybridized bonds. The electrical and optical properties of ta-C/ITO prepared on glass substrates were examined as a function of the thickness of the ta-C layer by Hall measurement (HMS-4000AM, Ecopia, Korea) and a UV/visible spectrometer (UV 540 spectrometer, Unicam, Japan). The surface morphology of the ta-C/ITO anode was examined with different ta-C thicknesses using field-emission scanning electron microscope (FE-SEM; JSM-7600F, JEOL, Japan). The work function of single ta-C films was measured by UPS. High-resolution transmission electron microscopy (HRTEM: JEM-2100F, JEOL, Japan) analysis was used to investigate the microstructure and interface region of the optimized ta-C/ITO anode used in the PSCs. Fast Fourier transform (FFT) images were obtained from a cross-sectional HRTEM specimen prepared through a focused ion beam (FIB) milling.

### 2.4. Fabrication and evaluation of PSCs and QDLEDs

**Figure 2.**
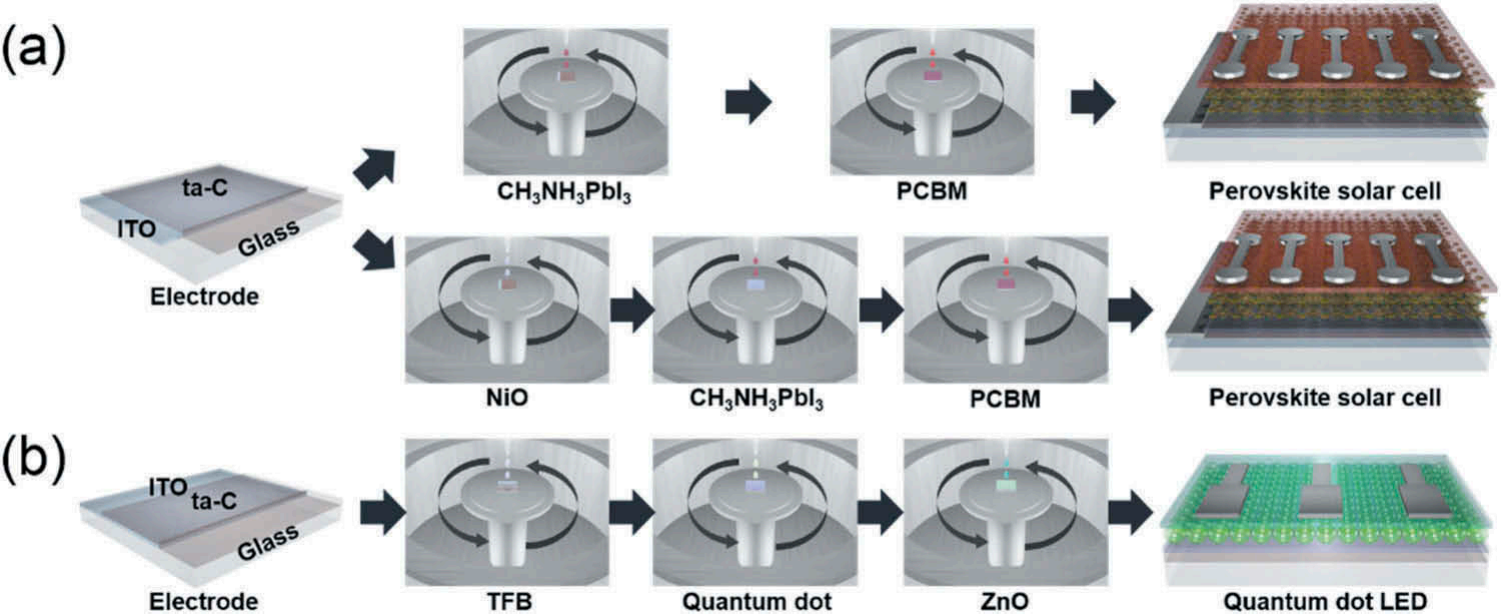
(a) Schematic of the PSCs' fabrication procedure using a single ta-C layer and a NiO/ta-C double layer as HTL. (b) Schematic of the QDLEDs' fabrication procedure on a patterned ta-C/ITO anode.

Figure 2 shows a schematic diagram of PSC and QDLED fabrication on the ta-C/ITO anode involving spin coating. Figure 2(a) shows a schematic diagram of the PSC fabrication process using a single ta-C layer and a NiO/ta-C double layer as HTL. To demonstrate the feasibility of ta-C film as HTL, PSCs were fabricated and evaluated using the ta-C film as a function of the thickness deposited on ITO anodes. The ta-C/ITO anodes were separately cleaned using deionized water, acetone, isopropanol, and ethanol in an ultrasonic cleaner for 15 min each to remove any potential organic and inorganic residue. Then, the ta-C/ITO anodes underwent UV/ozone treatment for 10 min before the deposition of the CH_3_NH_3_PbI_3_ or NiO layer. The NiO precursor in anhydrous alcohol was spin-coated at 4000 rpm for 40 s and the NiO films were annealed at 280°C for 60 min. The perovskite active layer CH_3_NH_3_PbI_3_ was coated onto the ta-C layer with a consecutive two-step spin coating where, in the second step, anhydrous chlorobenzene was dropped on the center of the substrate. The films were annealed at 100°C for 40 min to remove the solvent. Continuously, an electron transport layer, PCBM in chlorobenzene, was deposited on the perovskite active layer by spin coating at 1000 rpm for 60 s. Finally, 3 nm BCP and 100 nm Ag cathodes were formed successively using a thermal evaporator with a dumbbell-shaped metal shadow mask. The J-V curves were measured using simulated AM 1.5 G irradiation (100 mWcm^−2^), which was calibrated with a standard silicon photodiode under ambient condition. Figure 2(b) shows a schematic diagram of the QDLED fabrication process using a single ta-C layer as a HIL. First, poly[(9,9-dioctylfluorenyl-2,7-diyl)-*co*-(4,4A-(*N*-(4-*sec*-butylphenyl))diphenyla-mine)] (TFB, Lumtec) were mixed in *p*-xylene (1 wt %) and spin-coated onto the ta-C surface at 3000 rpm for 30 s, then annealed at 180°C for 30 min. Next, colloidal CdSe/ZnS QDs (NSQDs-HOS, Nanosquare) was spin-coated as an emissive layer (EML) and dried under ambient conditions. The substrate was then dipped into a 20 mL methanol solution in 1,7-diaminoheptane and subsequently rinsed with isopropanol and annealed at 180°C for 30 min to cross-link the QDs. The ZnO solution (N10, Avantama) was spin-coated as an ETL onto the QD surface at 2000 rpm for 60 s and annealed at 180°C for 30 min. Finally, 130 nm of Al cathode was deposited onto the ZnO ETL through a patterned shadow mask using a thermal evaporator. At this point, the electroluminescence (EL) characteristics of the device were measured using a conventional current–voltage–luminance (I-V-L) measurement system (McScience, M6100).

## 3. Results and discussion

**Figure 3.**
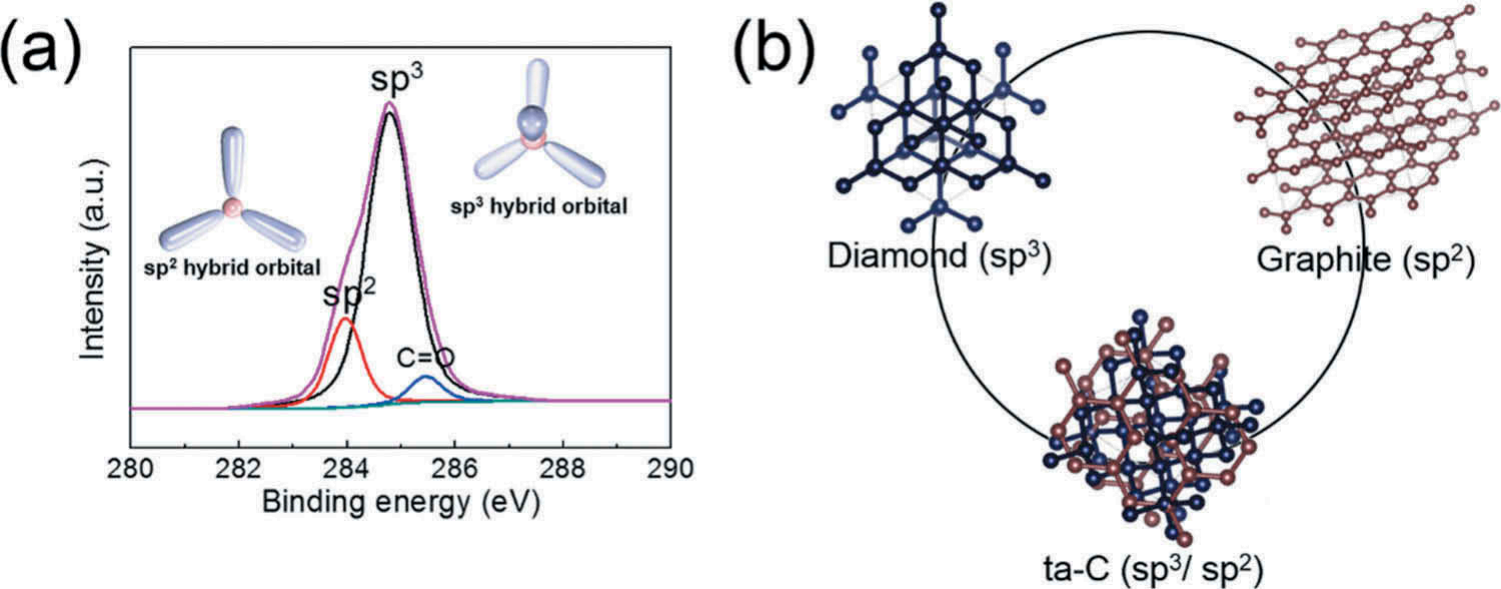
(a) XPS spectrum of a ta-C film. The inset schematic shows an sp^2^ hybrid orbital and sp^3^ hybrid orbital. (b) Schematic presentation of diamond (sp^3^) structure, graphite (sp^2^) structure, and ta-C (sp^3^/sp^2^) structure in which diamond and graphite structures are combined.

XPS survey measurements have been performed on the as-deposited ta-C film in order to evaluate the various bonding states of carbon in (Figure 3(a)). The results suggest that the mechanical, optical, and electrical properties of the ta-C films are significantly governed by the type of chemical bonding (hybridization) of carbon atoms. As shown in Figure 3(a), carbon atoms have graphite-like (sp^2^ hybridization) and diamond-like (sp^3^ hybridization) hybridizations, with a negligible amount of sp^1^ hybridization [53]. The sp^2^ hybridization is governed by the weak π-π bonding and determines the electrical and optical properties of the film. By contrast, sp^3^ hybridization occurs due to the strong σ-σ bonding of carbon atoms and offers the mechanical properties to the films. As shown in Figure 3(a), the C1*s* spectral peak is closer to 284.8 eV, indicating a higher number of sp^3^ bonds within the film, which can be calculated as approximately 77.69% (sp^2^ bonds: approximately 17.47%) from the Gaussian peak area ratio. The peaks located at 283.9, 284.7, and 285.4 eV are assigned to C=C sp^2^ hybridized carbon in graphite-like carbon-carbon structure, C-C sp^3^ hybridized carbon atoms in diamond-like carbon-carbon structure, and C=O bonding between carbon and oxygen, respectively [60]. Figure 3(b) shows the diamond structure of the sp^3^ hybridized bond, the graphite structure of the sp^2^ hybridized bond, and the ta-C structure of the sp^3^/sp^2^ hybridized bond. The results suggest that the combination of diamond-like mechanical properties and tunable electrical and optical properties of ta-C film makes it applicable to hole transport or injection layers for PSCs and QDLEDs, respectively.

**Figure 4.**
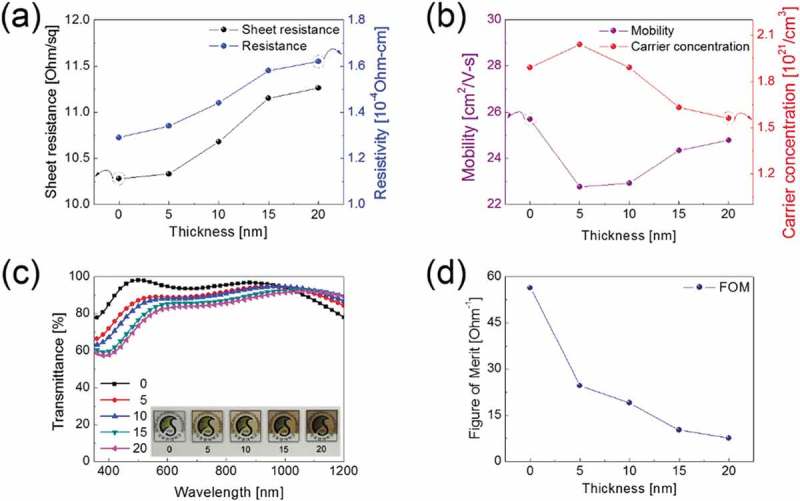
Hall measurement results of ta-C/ITO anodes with different ta-C thicknesses; 0, 5, 10, 15, 20 nm (a) Resistivity, sheet resistance, (b) Mobility and carrier concentration. (c) Optical transmittance and (d) Figure of merit (FoM = T^10^/R*_sh_*) of ta-C/ITO anodes as a function of ta-C thickness. Inset pictures in (c) show the color and transparency of the ta-C coated ITO anodes.

Figure 4(a,b) shows the Hall measurement results obtained from a ta-C/ITO anode with different ta-C thicknesses. With increasing ta-C film thickness, the sheet resistance and resistivity of the ta-C/ITO film both slightly increased. However, the resistivity was mainly affected by the quality of the bottom ITO electrode, and the ta-C/ITO anodes exhibited a similar low sheet resistance and resistivity within a ta-C layer thickness range of 20 nm. As shown in Figure 4(b), the increased sheet resistance and resistivity of the ta-C/ITO film with increasing ta-C thickness could be attributed to a decrease of the carrier concentration. However, the sheet resistance of ta-C coated ITO anode is acceptable for the fabrication of PSCs and QDLEDs. Figure 4(c) shows the variation of the optical transmittance of the ta-C/ITO anodes with different ta-C thicknesses in the wavelength region between 400 and 1200 nm. As the thickness of the ta-C layers increased, the ta-C/ITO anode showed a slightly lower optical transmittance, particularly in the visible wavelength region between 400 and 800 nm, compared to the bare ITO anode without ta-C film; this was attributed to absorption in the ta-C layers as shown in Figure S1(a). The optical band gap can be calculated from the absorption spectrum using the Tauc relation﻿:

(1) $$\left(\alpha hv\right)=C{\left(hv-E_{g}\right)}^{n}$$

where C is a constant, α is the absorption coefficient, $E_{g}$ is the average bandgap of the material, and n depends on the type of transition (in the parabolic band structure, n = 2). The optical bandgaps of single ta-C layers with different thicknesses are shown in Figure S1(b). In addition, the optical transmittance of the ta-C/ITO anodes gradually decreases at 550 nm due to the gray color of the ta-C layer. By contrast, the transmittance of the ITO anodes with ta-C film (5, 10, 15, 20 nm) increases with the thickness in the wavelength range of 1000 ~ 1200 nm due to the decreased carrier concentration. The inset pictures in the figure show the colors and transparencies of the single ITO and ta-C/ITO anodes with different ta-C thicknesses. In order to determine the optimum ta-C thickness, we compared the figure of merit (FoM = T^10^/R*_sh_*) values [62], which were calculated from the average optical transmittance (T) and the sheet resistance (R*_sh_*), as shown in Figure 4(d). Due to the fact that the active layer of the PSCs absorbs visible light (400 ~ 800 nm) along with the visible emission of QDLEDs, the FoM value is calculated using the average optical transmittance (400 ~ 800 nm) in the visible wavelength region. In addition, the voltage losses of the PSCs and QDLEDs are affected by the sheet resistance of the transparent electrode. Therefore, a higher FoM value generally indicates the increased quality of the transparent electrodes. With these considerations, we determined the optimum ta-C thickness on the ITO electrode. Based on FoM calculation, we found that the 5-nm thick ta-C layer had the highest FoM value of 24.6 Ω^−1^. With the optimized thickness of the ta-C layer (5 nm), the ta-C/ITO anode showed a sheet resistance of 10.33 Ω^−2^ and an optical transmittance of 88.97% at a wavelength of 550 nm.

**Figure 5.**
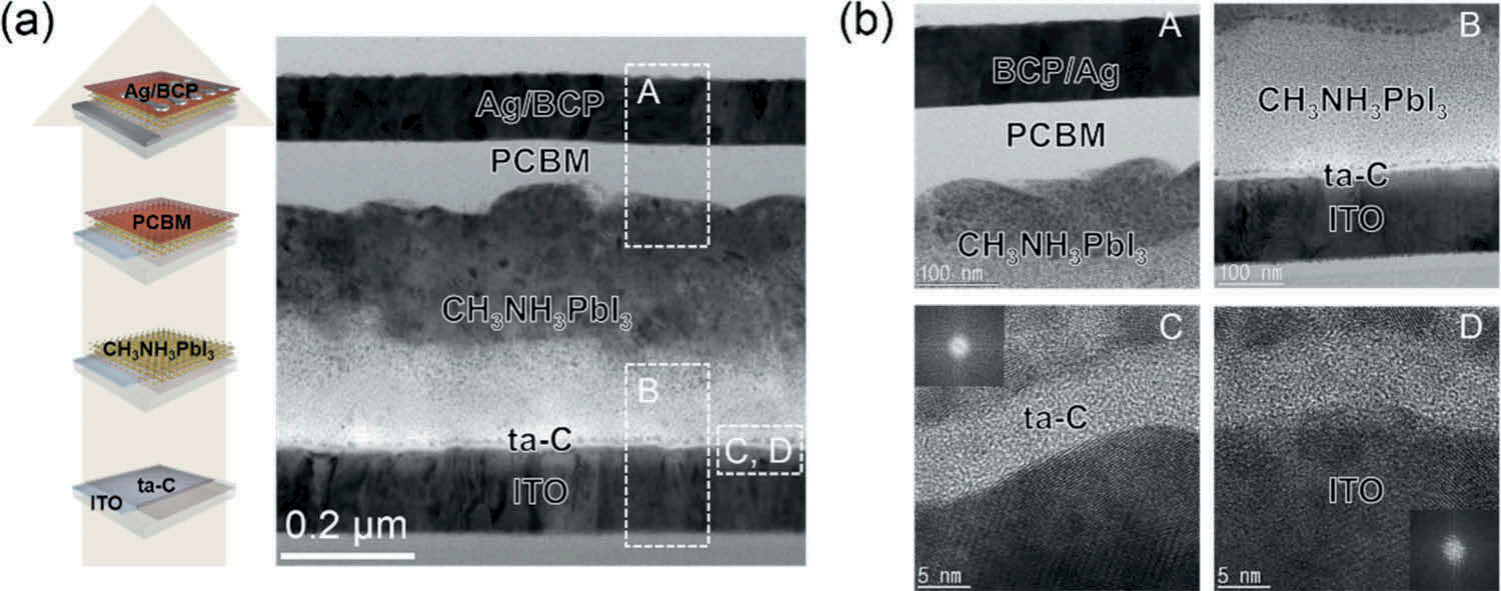
(a) Schematic PSC fabrication process on the ta-C/ITO anode and cross-sectional TEM image obtained from PSC with the ta-C/ITO anode. (b) Enlarged TEM images obtained from A (Ag/BCP/PCBM/CH_3_NH_3_PbI_3_ interface), B (CH_3_NH_3_PbI_3_/ta-C/ITO interface), C (ta-C), and D (ITO), respectively.

Figure 5(a) shows a cross-sectional TEM image of a PSC manufactured using a ta-C/ITO anode with the structure glass/ITO/ta-C/CH_3_NH_3_PbI_3_/PCBM/BCP:Ag. The well-distinguished interface between each layer in the cross-sectional TEM image indicates that no interdiffusion occurred. In addition, it shows uniform and smooth coating of the ta-C layer HTL on the ITO anode without any protrusions or hillocks. The square dotted outline enlarged images in Figure 5(b) demonstrate the smoothness of the interface. The BCP:Ag cathode layer in the ‘A’ region was in good contact with the PCBM layer without a diffuse interface, and the electron transport layer PCBM was in good contact with the CH_3_NH_3_PbI_3_ layer. The 3-nm thick BCP layer acted as a hole blocking and electron transport layer from PCBM to the Ag cathode in the PSCs. In addition, an enlarged image is shown of the 400-nm thick CH_3_NH_3_PbI_3_ active layer in the ‘B’ region. As shown in the image, there is good contact between the CH_3_NH_3_PbI_3_ active layer and the ta-C HTL without interdiffusion. The stable interface between the CH_3_NH_3_PbI_3_ layer and ta-C layer ensured facile hole transport and collection from the CH_3_NH_3_PbI_3_ layer to the bottom ITO anode. In addition, Figure S3(a) shows XRD patterns of CH_3_NH_3_PbI_3_ films coated on the NiO/ITO and the ta-C (5, 10, 15, 20 nm)/ITO substrates. Irrespective of the buffer layer, the CH_3_NH_3_PbI_3_ films showed identical crystalline structures. In the ‘C’ region, a cross-section TEM image and fast Fourier transform (FFT) pattern was obtained from the ta-C layer. The enlarged ta-C layer showed a completely disordered structure. In addition, the FFT showed a diffused pattern, indicating the amorphous structure of the ta-C layer. The TEM images indicate that the morphology of ta-C HTL well follows the morphology of the ITO anode. Further, FE-SEM images in Figures S2(a–d) show the amorphous surfaces of ta-C layers with different thicknesses deposited on ITO anodes. The ITO film below the ta-C layer region in ‘D’ shows a well-developed bixbyite structure with the (222) preferred orientation. The cross-section TEM image showed the vertically well-developed columnar structure of the ITO anode. The strong spots in FFT also indicate the polycrystalline structure of the ITO anode.

**Figure 6.**
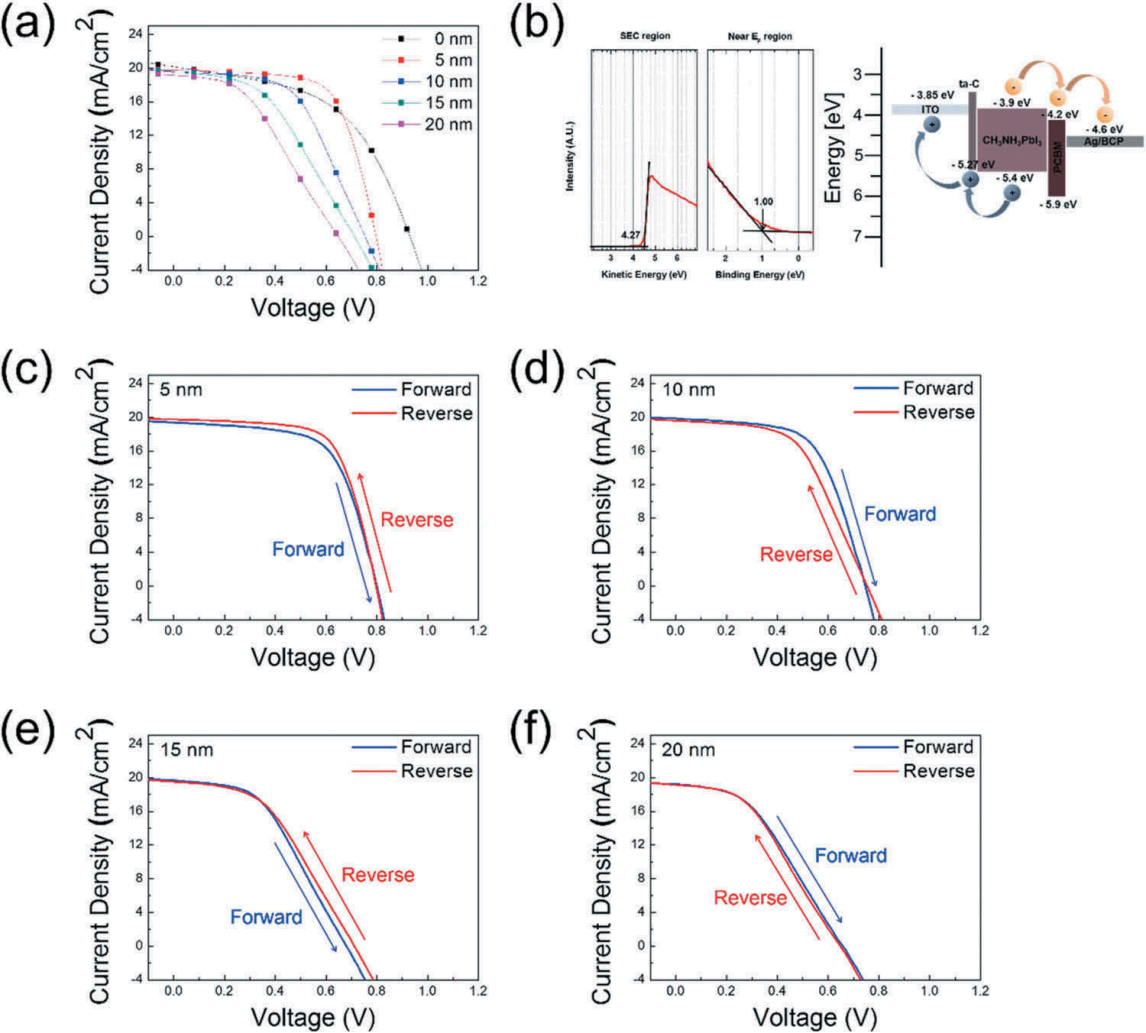
(a) Current density–voltage (J-V) curves of PSCs fabricated on the ta-C/ITO anodes with increasing ta-C thickness. (b) UPS spectrum used to determine the work function and valence band maximum (VBM) of the ta-C film and the energy band diagram of the PSCs. Current density–voltage (J-V) curves for forward and reverse scans (74 mV/s) of the solar cell with different ta-C thicknesses – (c) 5 nm, (d) 10 nm, (e) 15 nm, (f) 20 nm.

Figure 6(a) shows the current density–voltage (J-V) curves for PSCs using different thicknesses of the single ta-C layer, and the photovoltaic parameters are summarized in Table 1. The PSC fabricated on ITO anode with NiO HTL showed a power conversion efficiency (PCE) of 9.63%. The PCE of PSC fabricated on the ta-C (5)/ITO anode was 10.53%, slightly higher than that of the NiO/ITO anode. With increasing ta-C thickness, the PCE of PSCs decreased. Due to the gradual decrease in optical transmittance at a wavelength of 550 nm of the ta-C layer, the exciton generation of the perovskite active layer decreased. This affects J*_SC_* but V*_OC_* and FF decrease also. The PSC with ta-C 5-nm-thick layer exhibited a short circuit current density (J*_SC_*) of 19.73 mA/cm^2^, an open-circuit voltage (V*_OC_*) of 0.79 V, and a fill factor (FF) of 66.99%, leading to the highest PCE of 10.53%. Although PSC with 5-nm thick ta-C showed lower J*_SC_* and V*_OC_* values than NiO, its FF value was higher than that of PSC with the NiO/ITO anode. The FF value of a solar cell is mainly influenced by its shunt resistance (*R_SH_*) and series resistance (*R_S_*). Higher *R_SH_* and lower *R_S_* values are required to achieve ideally high FF in a solar cell. Therefore, we measured R*_SH_* and R*_S_* of the PSCs and summarized them in Table 1. From the fitting of the J-V curves, the PSC with ta-C HTL had higher *R_SH_* (1.09k Ω cm^2^) and lower *R_S_* (3.30 Ω cm^2^) than PSC with NiO HTL. As a result, the significant difference in series and shunt resistance can explain the improvement of FF from 51.34% to 66.99% as well as the improvement of PCE from 9.63% to 10.53%. The device statistics (open circuit voltage, short circuit current, fill factor, and PCE) with different ta-C thicknesses (5, 10, 15, 20 nm) are shown in Figure S4(a–d). Through UPS analysis, we measured the work function and valence band maximum (VBM) of ta-C films to identify the hole transport mechanism at the interface between HTL and the CH_3_NH_3_PbI_3_ active film. Figure 6(b) shows the spectrum used to determine the work function and VBM of the 5-nm thick ta-C film obtained from UPS analysis. The work function (Ф) of the film was evaluated via Ф = *hv* – *ΔE*, where *hv* is the photon energy (21.22 eV) and *ΔE* is determined based on the distance of the binding energy between the secondary electron emission cutoff (SEC) edge in the UPS spectra [63]. The work function and VBM of the 5nm thick ta-C film were respectively calculated to be 4.27 and 5.27 eV versus vacuum. The VBM of the single ta-C film (5.27 eV) as a hole transport layer was lower than that of the CH_3_NH_3_PbI_3_ film VBM (5.40 eV) as an active layer. An energy band diagram illustration of the PSCs ta-C/ITO anode applied to PSCs is shown in Figure 6(b). The PSC with ta-C HTL shows the hole transport process at the interface from the CH_3_NH_3_PbI_3_ layer and ITO layer. Because the ta-C layer has an appropriate VBM level, photogenerated holes were able to pass from the active layer to anode through the ta-C layer. Therefore, it can be seen that the ta-C layer plays a very important role as an HTL by operating the PSC. The hysteresis effect in PSCs was mainly related to the collection of excess carrier, defects in materials, ion movement caused by polarization, or ferroelectric effects [64]. The J-V curves show hysteresis of the PSCs depending on the scan direction and speed of the voltage during J-V measurements. Typically, reliable solar cell J-V measurements should exhibit coincident curves for both the forward and reverse scans. As shown in Figure 6(c–f), the J-V curves of PSCs performing with different ta-C thicknesses (5, 10, 15, 20 nm) showed a small hysteresis with a difference of under 1% in the efficiency between the forward scan and reverse scan (74 mV/s) under a 1 sun illumination condition of AM1.5G. Table S1 presents a summary of the photovoltaic parameters measured with forward and reverse scan direction for the best-performing solar cells with different ta-C thicknesses.

**Table 1.** Photovoltaic parameters derived from current–voltage (J-V) measurements of PSCs fabricated on the ta-C (0 nm; using NiO)/ITO anode and the ta-C (5, 10, 15, 20 nm)/ITO anodes.

Thickness [nm]	J*_SC_* [mA/cm^2^]	V*_OC_* [V]	FF [%]	PCE [%]	*R_SH_* [Ω cm^2^]	*R_S_* [Ω cm^2^]
0	20.19	0.93	51.34	9.63	0.21k	11.66
5	19.73	0.79	66.99	10.53	1.09k	3.30
10	19.58	0.75	54.58	8.05	0.64k	6.36
15	19.48	0.71	44.68	6.18	0.42k	10.11
20	19.09	0.65	40.86	5.05	0.39k	17.49

**Figure 7.**
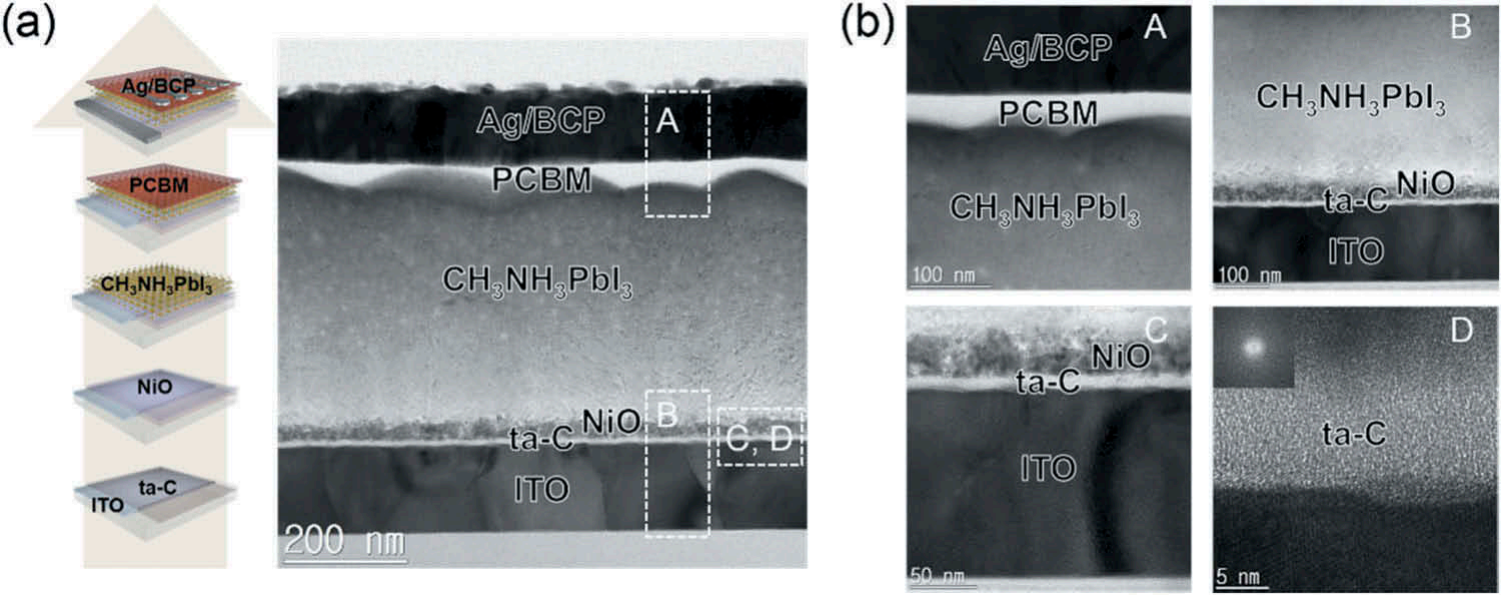
(a) Schematic PSC fabrication process on the double HTL of ta-C and NiO and cross-sectional TEM image obtained from PSC with the NiO/ta-C/ITO anode. (b) Enlarged TEM images obtained from A (Ag/BCP/PCBM/CH_3_NH_3_PbI_3_ interface), B (CH_3_NH_3_PbI_3_/NiO/ta-C/ITO interface), C (NiO/ta-C/ITO), and D (ta-C), respectively.

We also fabricated PSCs with NiO and ta-C double HTL as shown on the left side of Figure 7(a). On the ta-C (5 nm)/ITO and ta-C(10 nm)/ITO sample, we fabricated PSCs identical to the reference PSCs. The right side of Figure 7(a) shows a cross-sectional TEM image of a PSC with ta-C and NiO double HTL. The PSC had the following device structure: glass/ITO/ta-C/NiO/CH_3_NH_3_PbI_3_/PCBM/BCP:Ag. The enlarged square dotted outline images in Figure 7(b) show the quality of the interfaces in the PSCs. The BCP:Ag cathode layer in the ‘A’ region was in good contact with the PCBM layer without any interdiffusion. In addition, the electron transport layer PCBM was in good contact with the CH_3_NH_3_PbI_3_ layer. The 3-nm thick BCP layer acted as a hole blocking and electron transport layer from PCBM to the Ag cathode in the PSCs. In addition, an enlarged image of the 400-nm thick CH_3_NH_3_PbI_3_ active layer in the ‘B’ region is shown, where it can be seen that there is a sharp interface between the CH_3_NH_3_PbI_3_ active layer and the NiO hole extraction layer. In the ‘C’ region, the double hole transport layers NiO/ta-C are well distinguished with different contrast. The solution-processed NiO nanoparticle layer was in good contact with the FCVA-coated ta-C layer. The hole extraction and electron blocking of the NiO layer, along with the enhancement of hole transport in the ta-C layer, improved the hole extraction and transport from the CH_3_NH_3_PbI_3_ layer to the bottom ITO anode. In the ‘D’ region, the ta-C layer is a completely amorphous structure, as expected from the FFT pattern shown in the inset.

**Figure 8.**
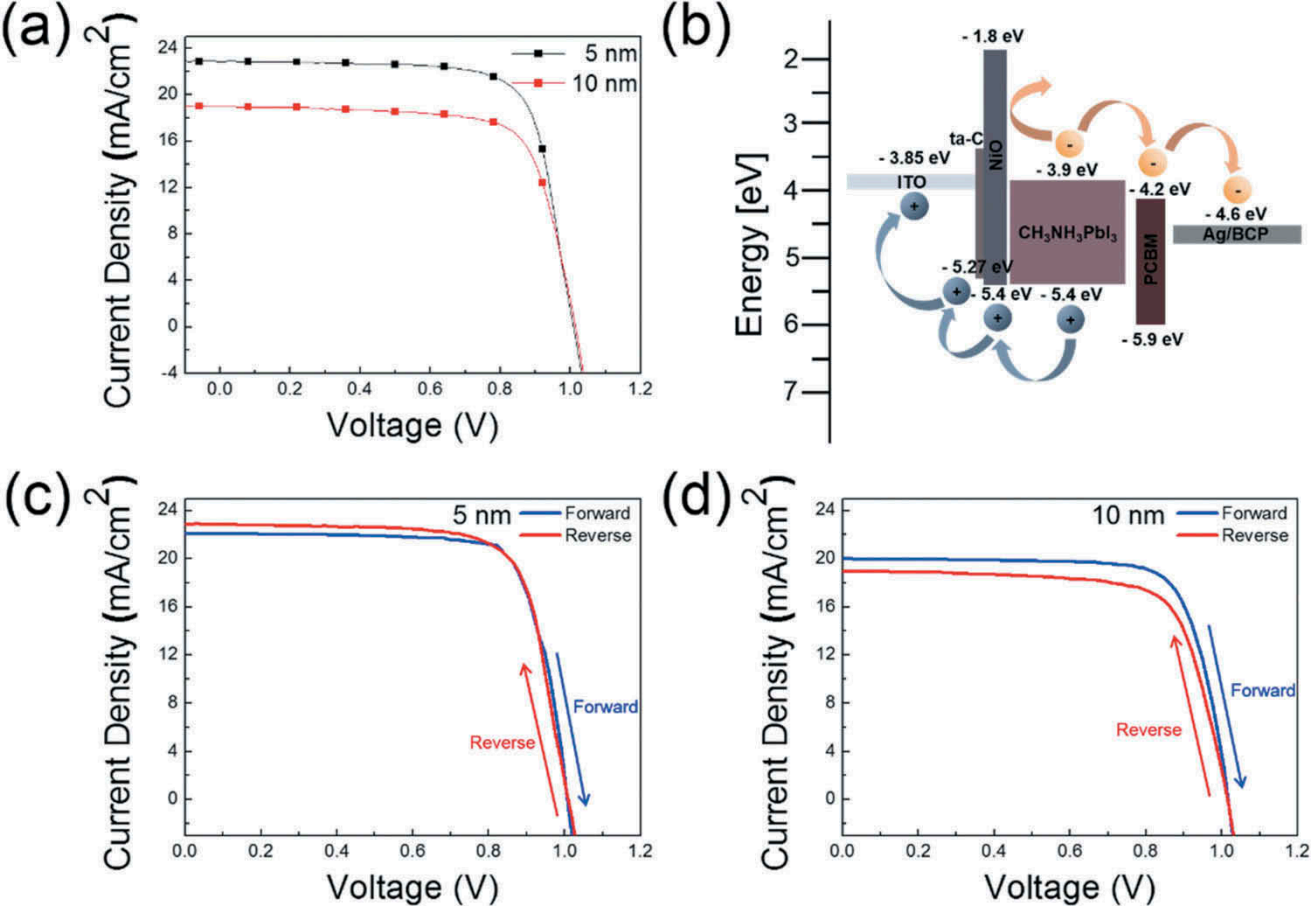
(a) Current density–voltage (J-V) curves of PSCs fabricated on the double HTL of ta-C (5, 10 nm) and NiO. (b) Energy band diagram of the PSCs with double HTL of ta-C and NiO. Current density–voltage (J-V) curves for forward and reverse scans (74 mV/s) of solar cells with ta-C and NiO double HTL; (c) ta-C 5 nm, (d) ta-C 10 nm.

Figure 8(a) shows the current density–voltage (J-V) curves of the PSC when the NiO layer is additionally applied to ta-C (5 and 10 nm), and the measured photovoltaic parameters that were used are summarized in Table 2. The PSCs fabricated by applying NiO and ta-C as a double HTL increased J*_SC_* compared to a single ta-C HTL. The PSC with ta-C (5 nm)/NiO exhibited a power conversion efficiency of 17.21% with a V*_OC_* of 1.01 V, J*_SC_* of 22.86 mA/cm^2^, and FF of 74.65%. The PSCs with ta-C (10 nm)/NiO showed a PCE of 14.05%, with a significant decrease in both the J*_SC_* (from 22.86 to 18.97 mA/cm^2^) and FF (from 74.65% to 73.13%). The device statistics (open circuit voltage, short circuit current, fill factor and PCE) with NiO/ta-C (5, 10 nm) double HTL are shown in Figure S5(a–d). Figure 8(b) shows an energy band diagram illustration of the NiO/ta-C/ITO anode applied to PSCs. When solar light is irradiated, excitons are generated in the perovskite active layer, and the holes generated by the exciton separation move to the ITO layer through the NiO and ta-C layer. In the energy band diagram of PSC, it can be seen that NiO functions as both a hole extraction/transport and electron blocking layer. Due to the essentially Ohmic contact between NiO and CH_3_NH_3_PbI_3_, a large Schottky barrier is not formed. The VBM of NiO is ~0.4 eV below the Fermi level (E_f_ = 5.0 eV), and with a NiO band-gap of ~3.6 eV, the conduction band energy is ~1.8 eV [65]. In addition, the photogenerated holes were able to pass through the ta-C layer due to the VBM (5.27 eV) of the ta-C layer being similar to that of the NiO (5.4 eV). Because a large number of hole carriers flowed into the ITO anode prior to recombination, the PSCs have a high FF value. Therefore, the NiO/ta-C double HTL improves the solar cell performance by effectively enhancing the hole extraction, transport, and electron blocking and suppressing the charge recombination. As a result, we showed the possibility of applying the coated ta-C film to the HTL of the PSCs by using the FCVA process. Figure 8(c,d) showed J-V curves measured via forward and reverse bias sweep for one of the best-performing solar cells with NiO/ta-C (5, 10 nm) double HTL. Irrespective of the ta-C thickness, the best performing PSC showed a small hysteresis with a difference of under 1% in the efficiency between the forward scan and reverse scan (74 mV/s) under standard AM1.5G conditions. A summary of the photovoltaic parameters measured with forward and reverse scan direction for the best-performing solar cells with NiO/ta-C (5, 10 nm) double HTL was shown in Table S2.

**Table 2.** Photovoltaic parameters derived from current–voltage (J-V) measurements of PSCs fabricated on the double HTL of ta-C (5, 10 nm) and NiO.

Thickness [nm]	J*_SC_* [mA/cm^2^]	V*_OC_* [V]	FF [%]	PCE [%]	*R_SH_* [Ω cm^2^]	*R_S_* [Ω cm^2^]
5	22.86	1.01	74.65	17.21	3.32k	0.24
10	18.97	1.01	73.13	14.05	2.32k	4.79

**Figure 9.**
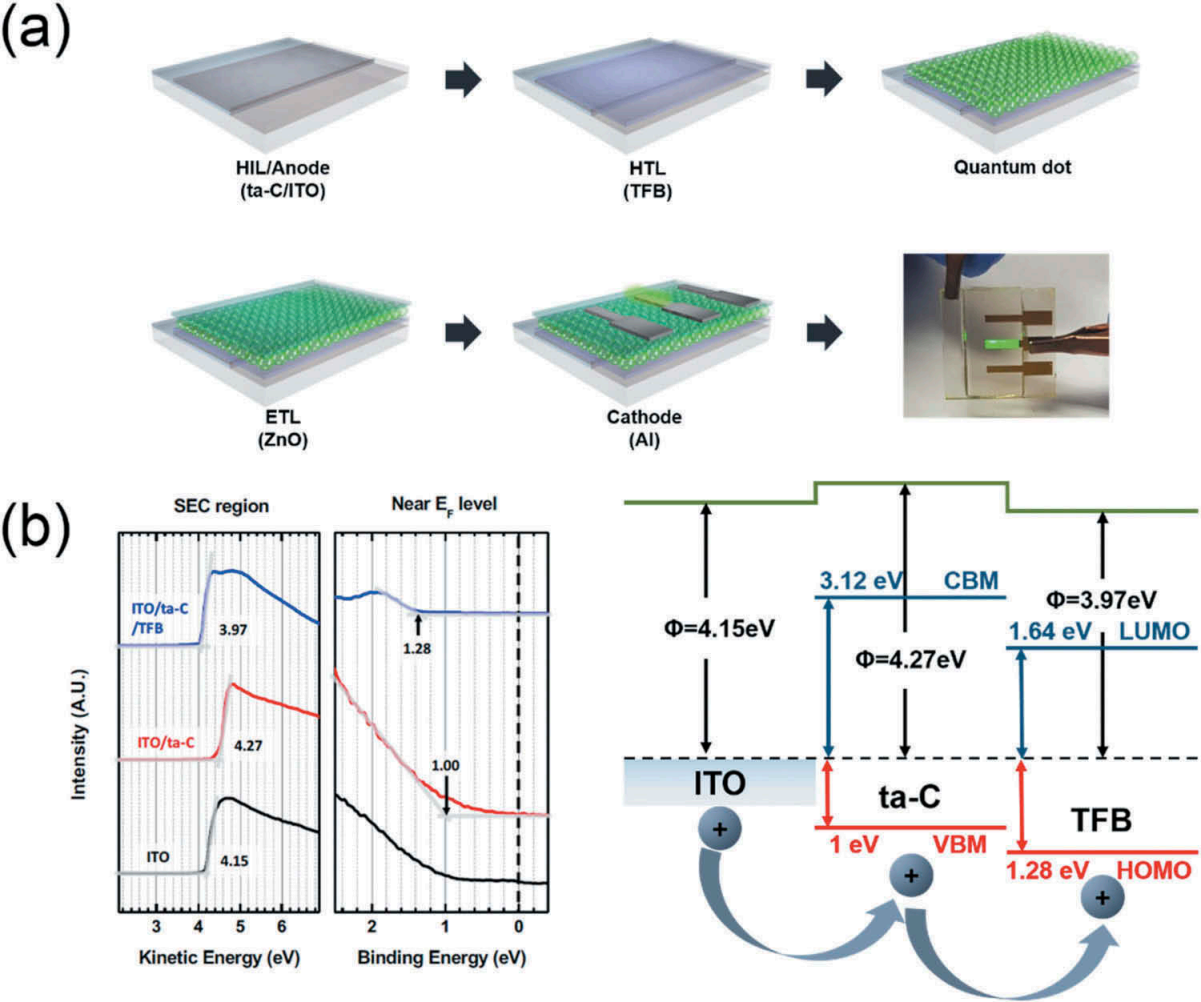
(a) Schematic fabrication processes of the QDLED on a patterned ta-C/ITO anode and the green light-emitting QDLED with a ta-C/ITO anode. (b) Measured UPS spectra secondary electron cutoff (SEC) and highest occupied molecular orbital (HOMO) or VBM region of TFB, ta-C, ITO. Interfacial energy level diagram of TFB/ta-C/ITO.

To show the potential of the FCVA processed ta-C as a hole injection layer (HIL), we fabricated QDLED with a patterned ta-C/ITO anode. Figure 9(a) is a schematic diagram showing the fabrication procedure of the QDLED on the ta-C applied as a HIL. The organic HTL, Green QDs-EML, and ETL were spin-coated on the patterned ta-C/ITO anode with different ta-C thicknesses (5, 10, 15 nm), and the LiF/Al cathodes were evaporated in a vacuum evaporator. The interfacial energy level diagrams of TFB/ta-C (5 nm)/ITO evaluated via UPS are summarized in Figure 9(b). The energy level diagrams were constructed by combining all of the information from the measured secondary electron cutoff (SEC) and the highest occupied molecular orbital (HOMO) or VBM region spectra. The work functions (Ф) of ta-C (5 nm) and TFB were measured as 4.27 and 3.97 eV, respectively. The VBM of ta-C (5 nm) on ITO was 1.0 eV and the HOMO level of TFB on ta-C (5 nm) was 1.28 eV from the Fermi level. To estimate the CBM and lowest unoccupied molecular orbital (LUMO) levels of ta-C (5 nm) and TFB, the bandgap of each film was evaluated through the Tauc plot of the UV/Vis absorption spectra data. The band gaps of ta-C (5 nm) and TFB were measured as 4.12 and 2.92 eV, respectively. The ionization energy (IE) of ta-C (5 nm) was 5.27 eV. Based on the VBM and HOMO level calculated from the vacuum level, the holes are injected into the HOMO level (5.27 eV) of TFB through the appropriate VBM level (5.25 eV) of ta-C (5 nm), as shown in the energy level diagram. Therefore, the ta-C (5) film could play a significant role in facilitating the hole injection from ITO into the emitting layer.

**Figure 10.**
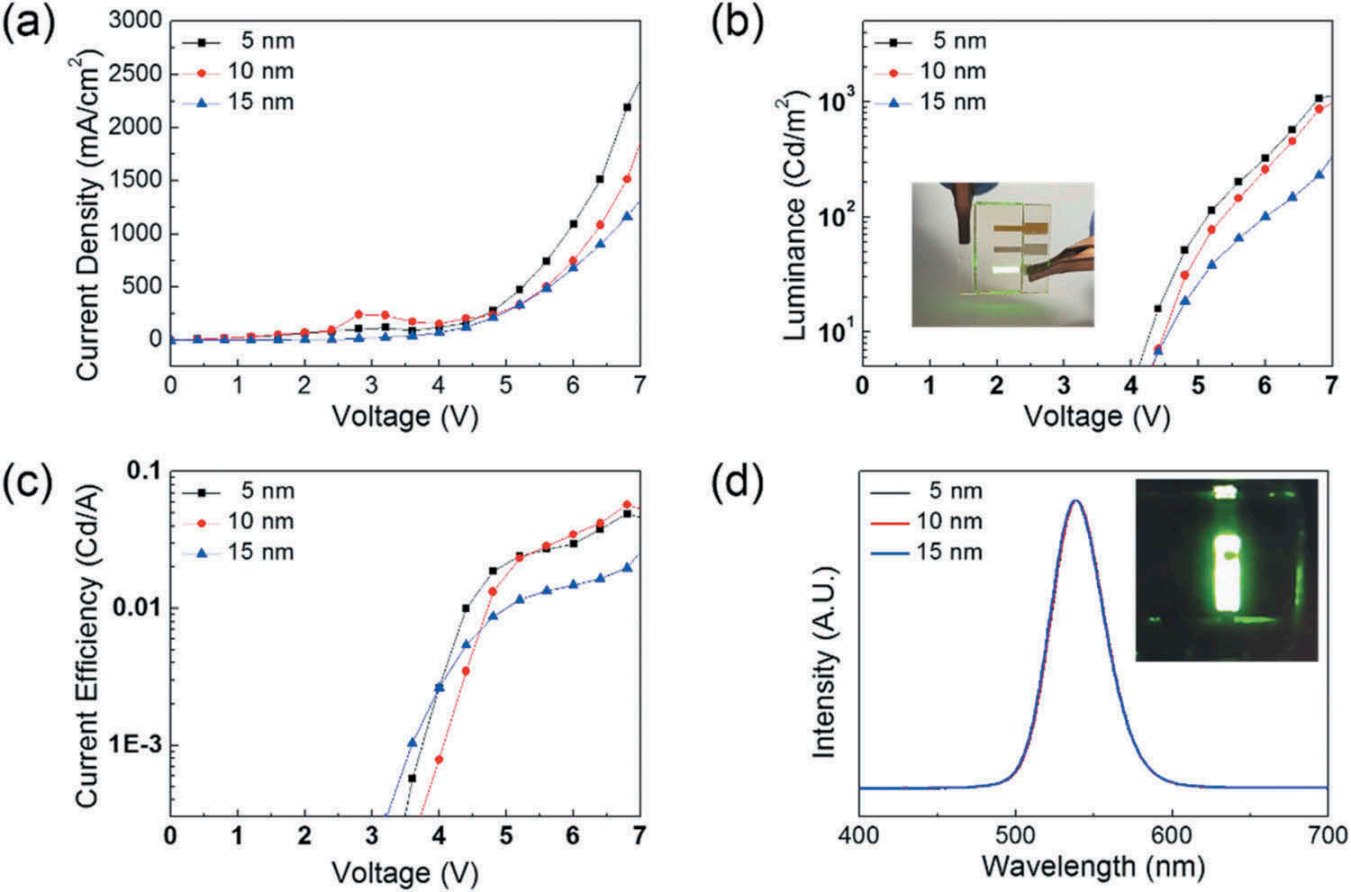
(a) Current density–voltage (J-V) curve, (b) luminance–voltage (L-V) curves, (c) Current efficiency–voltage curves, and (d) EL spectra of QDLEDs fabricated on ta-C/ITO anodes. The inset picture shows green light emission from the QDLED with the ta-C/ITO anode.

Figure 10 shows the current density–voltage (J-V) and luminance–voltage (L-V), current efficiency–voltage, and electroluminescence (EL) curve characteristics of the QDLEDs fabricated on the ta-C as a function of thickness. Figure 10(a) shows the current density versus the applied voltage for QDLEDs with different ta-C thicknesses. The maximum current density according to the applied voltage was 2705.2 mA/cm^2^ at 5 nm, 2219.2 mA/cm^2^ at 10 nm, and 1477.5 mA/cm^2^ at 15 nm. Figure 10(b) shows the L-V curves of the device with the same turn-on voltage of 4.0 V and a luminance of 1169.1 cd/m^2^ (at 5 nm thick), 1150.4 cd/m^2^ (at 10 nm thick), and 500.53 cd/m^2^ (at 15 nm thick). The maximum luminance was found in QDLEDs with 5-nm thick ta-C. The L-V curves of the QDLEDs with ta-C/ITO anodes also showed similar steep increases after onset. The inset picture shows an image of the light emission pixels from the QDLED with ta-C HIL processed by FCVA. In addition, as shown in Figure 10(c), the QDLED fabricated on the 5-nm thick ta-C layer showed a current efficiency of 0.043cd/A. Figure 10(d) shows a comparison of the electro-luminance spectra of the QDLEDs with different ta-C thicknesses. The spectra of green QDLEDs showed emission peaks at 532 nm. The inset picture shows the operation of the QDLEDs with a ta-C thickness of 5 nm.

## 4. Conclusions

In summary, we demonstrated the usage of ta-C/ITO anodes fabricated through the FCVA process as a hole transport or injection for PSCs and QDLEDs. The effects of using ta-C/ITO anodes as a function of the ta-C thickness are systemically investigated in terms of electrical, optical, morphological, and structural properties. Through XPS and UPS analyses of single ta-C films, we measured the moderate electrical properties (sp^3^/sp^2^ hybridized bond) and work function of ta-C layer to identify the hole transport or injection mechanism at the interface. The results showed that the performance of the PSC or QDLEDs is sensitive to the ta-C thickness. Under the optimized condition, the ta-C (5 nm)/ITO anodes exhibit the sheet resistance of 10.33 Ω^−2^, the resistivity of 1.34 × 10^−4^ Ω cm, and the optical transmittance of 88.97%. Due to the excellent optical transmittance and electrical conductivity along with the well-matched work function, the PSCs and QDLEDs with the ta-C/ITO anode were successfully operated. In the case of PSCs, the NiO/ta-C double HTL showed more efficient hole extraction and transport properties than the single NiO or ta-C layer. Based on the performance of the PSCs and QDLEDs with ta-C/ITO anodes, we suggest that ta-C film fabricated through an FCVA process acts as an effective hole transport or injection layer and can be applied in large area PSC and QDLEDs, unlike solution-processed PEDOT:PSS.

## References

[1] Wang D, Wright M, Elumalai NK, et al Stability of perovskite solar cells. Sol Energy Mater Sol Cells. 2016;147:255–275.

[2] Correa-Baena JP, Abate A, Saliba M, et al The rapid evolution of highly efficient perovskite solar cells. Energy Environ Sci. 2017;10:710–727.

[3] Asghar MI, Zhang J, Wang H, et al Device stability of perovskite solar cells – A review. Renewable Sustainable Energy Rev. 2017;77:131–146.

[4] Yan J, Saunders BR. Third-generation solar cells: a review and comparison of polymer: fullerene,hybrid polymer and perovskite solar cells. RSC Adv. 2014;4:43286–43314.

[5] Jamal MS, Bashar MS, Mahmud Hasan AK, et al Fabrication techniques and morphological analysis of perovskite absorber layer for high-efficiency perovskite solar cell: A review. Renewable Sustainable Energy Rev. 2018;98:469–488.

[6] Calió L, Kazim S, Grätzel M, et al Hole-transport materials for perovskite solar cells. Angew Chem Int Ed. 2016;55:14522–14545. 10.1002/anie.201601757 27739653

[7] Bakr ZH, Wali Q, Fakharuddin A, et al Advanced in hole transport materials engineering for stable and efficient perovskite solar cells. Nano Energy. 2017;34:271–305.

[8] Cao W, Xiang C, Yang Y, et al Highly stable QLEDs with improved hole injection via quantum dot structure tailoring. Nat Commun. 2018;9:2608. 29973590 10.1038/s41467-018-04986-z PMC6031613

[9] Mashford BS, Stevenson M, Popovic Z, et al High-efficiency quantum-dot light-emitting devices with enhanced charge injection. Nat Photonics. 2013;7:407–412.

[10] Yang Y, Zheng Y, Cao W, et al High-efficiency light-emitting devices based on quantum dots with tailored nanostructures. Nat Photonics. 2015;9:259–266.

[11] Cao F, Wang H, Shen P, et al High-efficiency and stable quantum dot light-emitting diodes enabled by a solution-processed metal-doped nickel oxide hole injection interfacial layer. Adv Funct Mater. 2017;27:1704278.

[12] Kim HM, Kim J, Jang J, et al Quantum-dot light-emitting diodes with a perfluorinated ionomer-doped copper-nickel oxide hole transport layer. Nanoscale. 2018;10:7281–7290. 29632918 10.1039/c7nr09671b

[13] Dai X, Zhang Z, Jin Y, et al Solution-processed, high-performance light-emitting diodes based on quantum dots. Nature. 2014;515:96–99. 25363773 10.1038/nature13829

[14] Xing G, Mathews N, Sun S, et al Long-range balanced electron-and hole-transport lengths in organic-inorganic CH_3_NH_3_PbI_3_. Science. 2013;342:344–347. 24136965 10.1126/science.1243167

[15] Stranks SD, Eperon GE, Grancini G, et al Electron-hole diffusion lengths exceeding 1 micrometer in an organometal trihalide perovskite absorber. Science. 2013;342:341–344. 24136964 10.1126/science.1243982

[16] Wolf SD, Holovsky J, Moon SJ, et al Organometallic halide perovskites: sharp optical absorption edge and its relation to photovoltaic performance. J Phys Chem Lett. 2014;5:1035–1039. 26270984 10.1021/jz500279b

[17] Yin WJ, Shi T, Yan Y, et al Unique properties of halide perovskites as possible origins of the superior solar cell performance. Adv Mater. 2014;26:4653–4658. 24827122 10.1002/adma.201306281

[18] Dong Q, Fang Y, Shao Y, et al Electron-hole diffusion lengths > 175 μm in solution-grown CH_3_NH_3_PbI_3_ single crystals. Science. 2015;347:967–970. 25636799 10.1126/science.aaa5760

[19] Im JH, Lee CR, Lee JW, et al 6.5% efficient perovskite quantum-dot-sensitized solar cell. Nanoscale. 2011;3:4088–4093. 21897986 10.1039/c1nr10867k

[20] Zhou H, Chen Q, Li G, et al Interface engineering of highly efficient perovskite solar cells. Science. 2014;345:542–546. 25082698 10.1126/science.1254050

[21] Yang WS, Noh JH, Jeon NJ, et al High-performance photovoltaic perovskite layers fabricated through intramolecular exchange. Science. 2015;348:1234–1237. 25999372 10.1126/science.aaa9272

[22] Saliba M, Matsui T, Seo JY, et al Cesium-containing triple cation perovskite solar cells: improved stability, reproducibility and high efficiency. Energy Environ Sci. 2016;9:1989–1997. 27478500 10.1039/c5ee03874j PMC4936376

[23] Yang D, Yang R, Wang K, et al High efficiency planar-type perovskite solar cells with negligible hysteresis using EDTA-complexed SnO_2_. Nat Commun. 2018;9:3239. 30104663 10.1038/s41467-018-05760-x PMC6089874

[24] Shen H, Duong T, Wu Y, et al Metal halide perovskite: a game-changer for photovoltaics and solar devices via a tandem design. Sci Technol Adv Mater. 2018;19:53–75.

[25] Zhang H, Toudert J Optical management for efficiency enhancement in hybrid organic-inorganic lead halide perovskite solar cells. Sci Technol Adv Mater. 2018;19:411–424. 29868146 10.1080/14686996.2018.1458578 PMC5974756

[26] National Renewable Energy Laboratory (NREL) [Internet] Golden (USA). 2019 [cited 2019 824]. Available from: https://www.nrel.gov/pv/cell-efficiency.html

[27] Shen H, Cao W, Shewmon NT, et al High-efficiency, low turn-on voltage blue-violet quantum-dot-based light-emitting diodes. Nano Lett. 2015;15:1211–1216. 25580801 10.1021/nl504328f

[28] Deng W, Fang H, Jin X, et al Organic-inorganic hybrid perovskite quantum dots for light-emitting diodes. J Mater Chem C. 2018;6:4831–4841.

[29] Song J, Li J, Li X, et al Quantum dot light-emitting diodes based on inorganic perovskite cesium lead halides (CsPbX_3_). Adv Mater. 2015;27:7162–7167. 26444873 10.1002/adma.201502567

[30] Malinkiewicz O, Yella A, Lee YH, et al Perovskite solar cells employing organic charge-transport layer. Nat Photonics. 2014;8:128–132.

[31] You J, Meng L, Song TB, et al Improved air stability of perovskite solar cells via solution-processed metal oxide transport layer. Nat Nanotechnol. 2016;11:75–81. 26457966 10.1038/nnano.2015.230

[32] Yang G, Tao H, Qin P, et al Recent progress in electron transport layers for efficient perovskite solar cells. J Mater Chem A. 2016;4:3970–3990.

[33] Cao J, Wu B, Chen R, et al Efficient, hysteresis-free, and stable perovskite solar cells with ZnO as Electron-transport layer: effect of surface passivation. Adv Mater. 2018;30:1705596. 10.1002/adma.201705596 29349858

[34] Li Z, Hu Y, Shen H, et al Efficient and long-life green light-emitting diodes comprising tridentate thiol capped quantum dots. Laser Photonics Rev. 2017;11:1600227.

[35] Zaiats G, Ikeda S, Kinge S, et al Quantum dot light-emitting devices: beyond alignment of energy levels. ACS Appl Mater Interfaces. 2017;9:30741–30745. 28841285 10.1021/acsami.7b07893

[36] Cho I, Jung H, Jeong BG, et al Multifunctional dendrimer ligands for high-efficiency, solution-processed quantum dot light-emitting diodes. ACS Nano. 2017;11:684–692. 27973766 10.1021/acsnano.6b07028

[37] Nam S, Oh N, Zhai Y, et al High efficiency and optical anisotropy in double-heterojunction nanorod light-emitting diodes. ACS Nano. 2015;9:878–885. 25565187 10.1021/nn506577p

[38] de Jong MP, van IJzendoorn LJ, de Voigt MJA, et al Stability of the interface between indium-tin-oxide and poly(3,4-ethylenedioxythiophene)/poly(styrenesulfonate) in polymer light-emitting diodes. Appl Phys Lett. 2000;77:2255–2257.

[39] van Duren JKJ, Loos J, Morrissey F, et al In-situ compositional and structural analysis of plastic solar cells. Adv Funct Mater. 2002;12:665–669.

[40] Kawano K, Pacios R, Poplavskyy D, et al Degradation of organic solar cells due to air exposure. Sol Energy Mater Sol Cells. 2006;90:3520–3530.

[41] Yang X, Zhang ZH, Ding T, et al High-efficiency all-inorganic full-colour quantum dot light-emitting diodes. Nano Energy. 2018;46:229–233.

[42] Yu Z, Sun L Inorganic hole-transporting materials for perovskite solar cells. Small Methods. 2018;2:1700280.

[43] Zheng X, Chen H, Li Q, et al Boron doping of multiwalled carbon nanotubes significantly enhances hole extraction in carbon-based perovskite solar cells. Nano Lett. 2017;17:2496–2505. 28287749 10.1021/acs.nanolett.7b00200

[44] Aitola K, Domanski K, Correa-Baena JP, et al High temperature-stable perovskite solar cell based on low-cost carbon nanotube hole contact. Adv Mater. 2017;29:1606398. 10.1002/adma.201606398 28229537

[45] Jeng JY, Chiang YF, Lee MH, et al CH3NH3PbI3 perovskite/fullerene planar-heterojunction hybrid solar cells. Adv Mater. 2013;25:3727–3732. 23775589 10.1002/adma.201301327

[46] Yan K, Wei Z, Li J, et al High-performance graphene-based hole conductor-free perovskite solar cells: schottky junction enhanced hole extraction and electron blocking. Small. 2015;11:2269–2274. 25641809 10.1002/smll.201403348

[47] Yang J, Lee J, Lee J, et al Mobility enhancement of hole transporting layer in quantum-dot light-emitting diodes incorporationg single-walled carbon nanotubes. Diamond Relat Mater. 2017;73:154–160.

[48] Neuville S, Matthews A A perspective on the optimisation of hard carbon and related coatings for engineering applications. Thin Solid Films. 2007;515:6619–6653.

[49] In JH, Kim YB, Hwang Y, et al Control of residual stress of tetrahedral amorphous carbon thin film deposited on dielectric material by filtered cathodic vacuum arc source by using mid-frequency pulse bias voltage. Surf Coat Technol. 2018;349:909–916.

[50] Polo MC, Andújar JL, Hart A, et al Preparation of tetrahedral amorphous carbon films by filter cathodic vacuum arc deposition. Diamond Relat Mater. 2000;9:663–667.

[51] Chhowalla M, Ferrari AC, Robertson J, et al Evolution of sp^2^ bonding with deposition temperature in tetrahedral amorphous carbon studied by raman spectroscopy. Appl Phys Lett. 2000;76:1419–1421.

[52] Waidmann S, Knupfer M, Fink J, et al Electronic structure studied of undoped and nitrogen-doped tetrahedral amorphous carbon using high-resolution electron energy-loss spectroscopy. J Appl Phys. 2001;89:3783–3792.

[53] Ishpal, Panwar OS, Kumar M, et al Effect of ambient gaseous environment on the properties of amorphous carbon thin films. Mater Chem Phys. 2011;125:558–567.

[54] Xu S, Flynn D, Tay BK, et al Mechanical properties and Raman spectra of tetrahedral amorphous carbon films with high sp^3^ fraction deposited using a filtered cathodic arc. Philos Mag B. 1997;76:351–361.

[55] Xu S, Cheah LK, Tay BK, et al Spectroscopic ellipsometry studies of tetrahedral amorphous carbon prepared by filtered cathodic vacuum arc technique. Thin Solid Films. 1998;312:160–169.

[56] Cheah LK, Shi X, Shi JR, et al Properties of nitrogen doped tetrahedral amorphous carbon films prepared by filtered cathodic vacuum arc technique. J Non-Cryst Solids. 1998;242:40–48.

[57] Miyajima Y, Henley SJ, Adamopoulos G, et al Pulsed laser deposited tetrahedral amorphous carbon with high sp^3^ fractions and low optical bandgaps. J Appl Phys. 2009;105:073521.

[58] Xu S, Tay BK, Tan HS, et al Properties of carbon ion deposited tetrahedral amorphous carbon films as a function of ion energy. J Appl Phys. 1996;79:7234–7240.

[59] Iyer A, Kaskela A, Novikov S, et al Effect of tetrahedral amorphous carbon coating on the resistivity and wear of single-walled carbon nanotube network. J Appl Phys. 2016;119:185306.

[60] Wang X, Zhao Y Study of electrical conductivity and microscosmic structure of tetrahedral amorphous carbon films doped by boron. Adv Mater Sci Eng. 2015;2015:1–6.

[61] Jang YJ, Kang YJ, Kitazume K, et al Mechanical and electrical properties of micron-thick nitrogen-doped tetrahedral amorphous carbon coating. Diamond Relat Mater. 2016;69:121–126.

[62] Haacke G New figure of merit for transparent conductors. J Appl Phys. 1976;47:4086–4089.

[63] Park Y, Choong V, Gao Y, et al Work function of indium tin oxide transparent conductor measured by photoelectron spectroscopy. Appl Phys Lett. 1996;68:2699–2701.

[64] Snaith HJ, Abate A, Ball JM, et al Anomalous hysteresis in perovskite solar cells. J. Phys Chem Lett. 2014;5:1511–1515. 10.1021/jz500113x 26270088

[65] Irwin MD, Bruce Buchholz D, Hains AW, et al P-type semiconducting nickel oxide as an efficiency-enhancing anode interfacial layer in polymer bulk-heterojunction solar cells. PNAS. 2008;105:2783–2787.

