# Supplementary material for: Tetrahedral amorphous carbon prepared filter cathodic vacuum arc for hole transport layers in perovskite solar cells and quantum dots LEDs
Source: Sci Technol Adv Mater. 2019 Nov 22;20(1):1118–30. doi: 10.1080/14686996.2019.1694841 (PMC6968577; doi:10.1080/14686996.2019.1694841)
Supplement: Supplemental Material [file TSTA_A_1694841_SM8953.pdf]

Supplementary information

A tetrahedral amorphous carbon film applied as a hole transport layer prepared by the filter cathode vacuum arc process for perovskite solar cells and quantum dots LEDs

Hae-Jun Seok[a], Yong-Jin Kang[b], Jongkuk Kim[b], Do-Hyeong Kim[c], Su Been Heo[d], Seong Jun Kang[d] and Han-Ki Kim[a,*]

[a]School of Advanced Materials Science and Engineering, Sungkyunkwan University, Suwon-si, Gyeonggi-do, Republic of Korea
[b]Surface Engineering Department, Implementation Research Division, Korea Institute of Materials Science (KIMS), Changwon-si, Republic of Korea
[c]Korea Electric Power Research Institute, Daejeon, Republic of Korea
[d]Department of Advanced Materials Engineering for Information and Electronics, Kyung-Hee University, Yongin-si, Gyeonggi-do, Republic of Korea

E-mail: hankikim@skku.edu (Prof. H.-K. Kim) *Tel: +82-31-290-7391 Fax: +82-31-290-7410;*

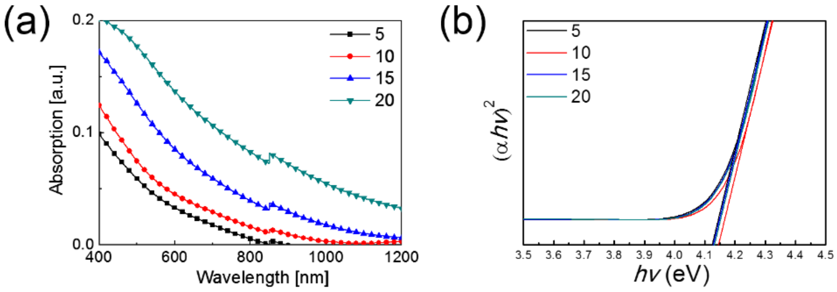

Figure S1. (a) Optical absorption and (b) optical bandgap of single ta-C film with different ta-C thickness; 0, 5, 10, 15, 20 nm.

Analysis of optical absorption and optical bandgap of single ta-C films. For the UV/visible spectrometer analysis, we measured the optical absorption and optical bandgap of single ta-C films with different thickness (5 ~ 20 nm) as shown in Figure S1(a). With the increase in ta-C thickness, the optical absorption increased in the visible wavelength region between 400 and 800 nm. The optical band can be calculated from the absorption spectrum using Tauc relation. We show the optical bandgap of single ta-C films as a function of thickness in Figure S1(b).

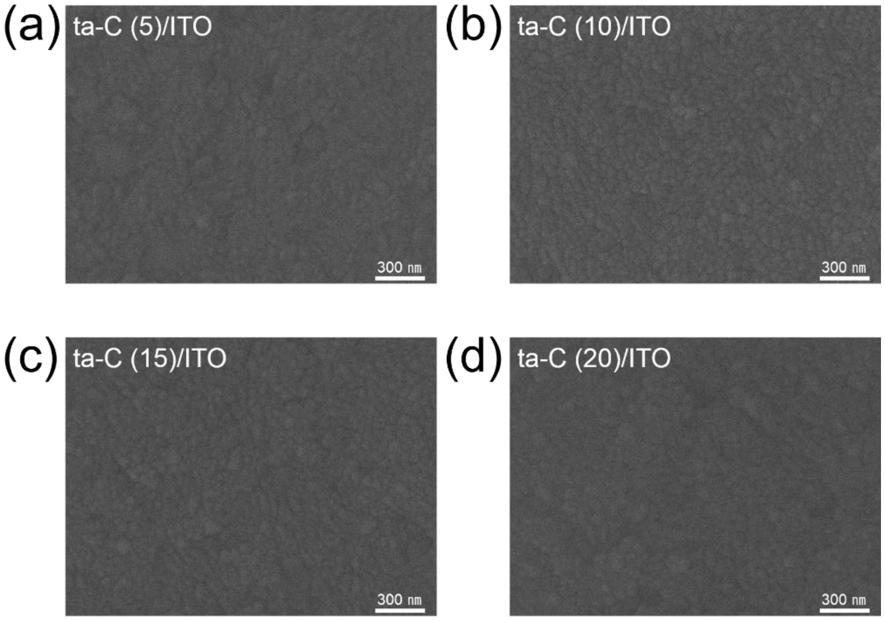

Figure S2. Surface FE-SEM images obtained from (a) ta-C (5 nm)/ITO, (b) ta-C (10 nm)/ITO, (c) ta-C (15 nm)/ITO, and (d) ta-C(20 nm)/ITO anodes.

Morphological properties of ta-C/ITO anodes. We show surface FE-SEM images ta-C deposited ITO electrodes prepared on glass substrates with increasing ta-C thickness as shown in Figure S2. The surface FE-SEM images of the ta-C films show a typical amorphous surface morphology without surface defects such as pin holes, cracks, and protrusions. Due to low process temperature of FCVA, the ta-C film exhibited a featureless surface morphology. It is noteworthy that the ta-C coating on the ITO anode did not affect on the surface morphology. The surface morphology of the ITO anode (Figure S2(a)-(d)) coated with ta-C layers from 5 nm to 20 nm thickness is completely covered by the amorphous surface of the ta-C layer.

Information Classification: General

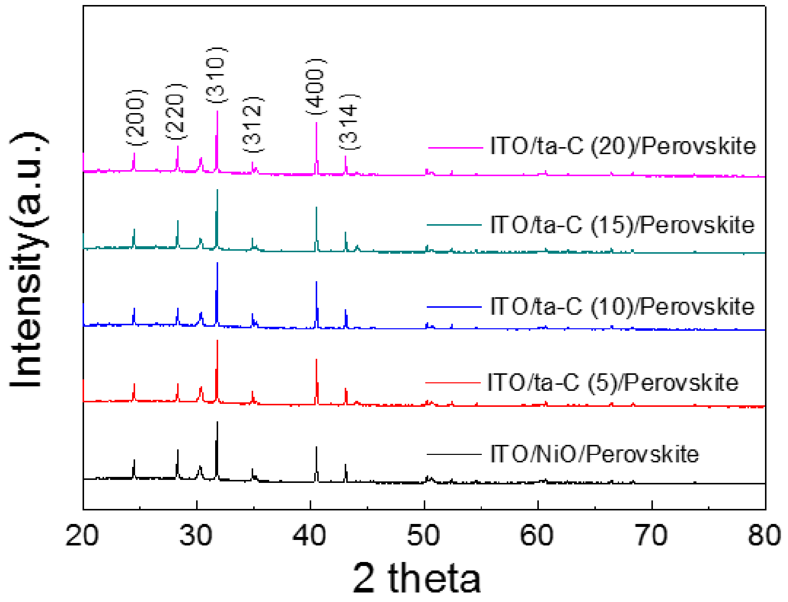

Figure S3. XRD patterns of perovskite on NiO/ITO and ta-C (5, 10, 15, 20 nm)/ITO substrates.

Crystalline properties of perovskite films. Figure S3 shows the XRD patterns of $CH_3NH_3PbI_3$ films deposited on NiO/ITO and ta-C (5, 10, 15, 20 nm)/ITO substrates. The XRD results show that the crystallinity of the $CH_3NH_3PbI_3$ perovskite films deposited on the NiO film and ta-C film was exactly identical irrespective of the buffer layer. Their clearly indicates that the ta-C buffer does not affect the microstructure of the $CH_3NH_3PbI_3$ perovskite active layer

Information Classification: General

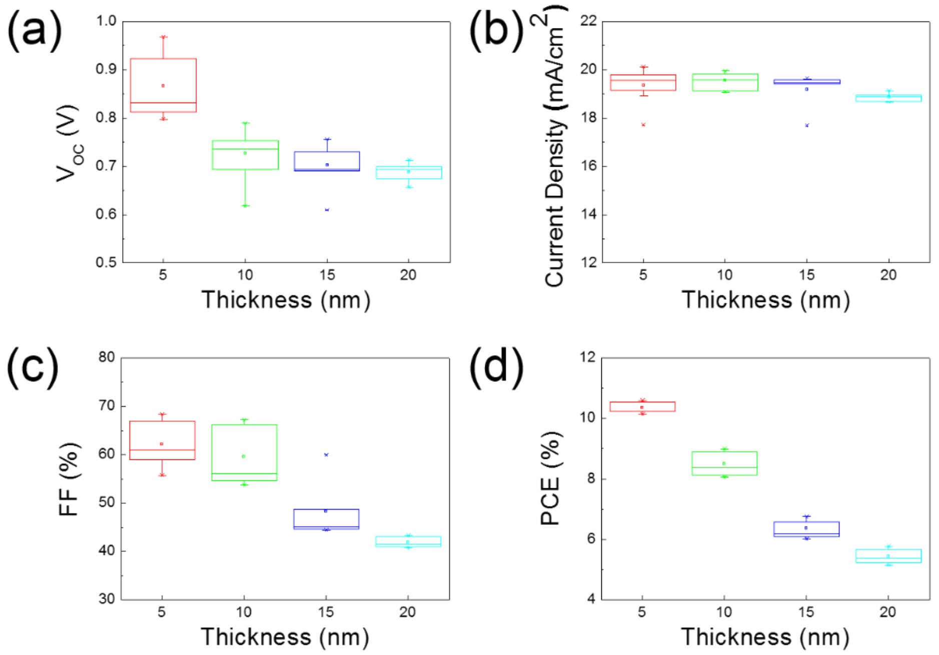

Figure S4. Box chart of photovoltaic parameters for PSCs prepared with different ta-C thicknesses. (a) V_{OC}, (b) J_{SC}, (c) FF, and (d) PCE.

The statistics chart of photovoltaic parameter for PSCs with different thicknesses of the ta-C layer. The statistical results of the photovoltaic parameters extracted from a series of PSCs are shown in Figure S4(a)-(d). Each box presents the parameter distribution of 10 devices under similar working condition. With increasing ta-C thickness, the performance metrics (V_{OC}, J_{SC}, FF, PCE) of PSCs decreased. Due to the gradually decrease in optical transmittance at a wavelength of 550 nm of the ta-C layer, the exciton generation of the perovskite active layer decreased. The statistical results further confirm similar trend to the best PSCs with different single ta-C thicknesses.

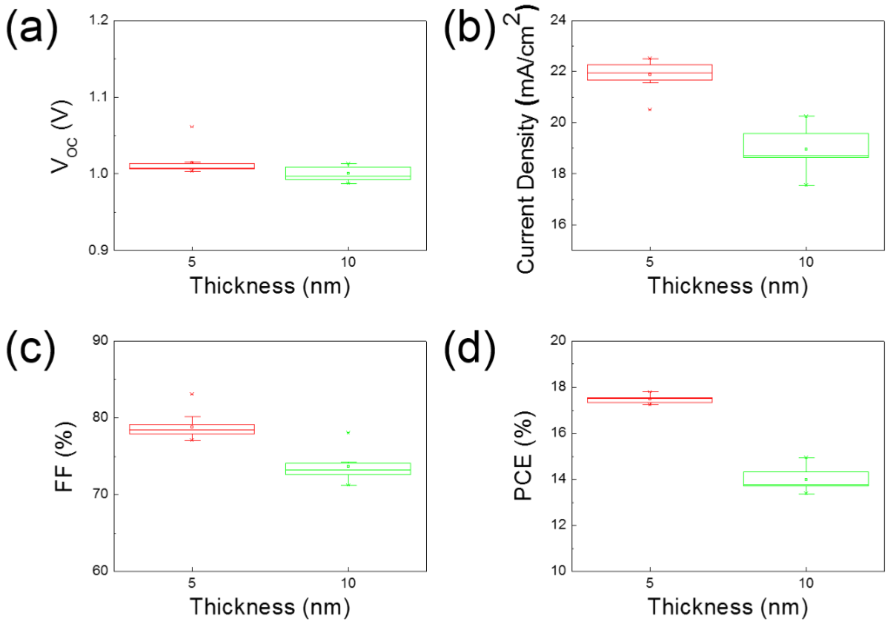

Figure S5. Box chart of photovoltaic parameters for PSCs prepared with ta-C (5, 10 nm) and NiO double HTL. (a) V_{OC}, (b) J_{SC}, (c) FF, and (d) PCE.

The statistics chart of photovoltaic parameter for PSCs with ta-C (5, 10 nm) and NiO double HTL. The statistical results of the photovoltaic parameters extracted from a series of PSCs are shown in Figure S5(a)-(d). Each box presents the parameter distribution of 10 devices under similar working condition. The PSC with ta-C (5)/NiO exhibited a PCE of 17.21% with a V_{OC} of 1.01 V, J_{SC} of 22.86 mA/cm^2, and FF of 74.65%. The PSCs with ta-C (10)/NiO showed a PCE of 14.05%, with a significant decrease in both the J_{SC} (from 22.86 to 18.97 mA/cm^2) and FF (from 74.65 to 73.13%). The statistical results of the photovoltaic parameters further confirm similar trend to the performance parameter of best PSCs with ta-C (5, 10 nm) and NiO double HTL.

Information Classification: General

Table S1. Photovoltaic parameters of PSCs fabricated on the ta-C/ITO anodes with different ta-C thicknesses measured with forward and reverse scan directions.

Thickness [nm]		J_{SC} [mA/cm^2]	V_{OC} [V]	FF [%]	PCE [%]
5	Forward	19.38	0.80	63.51	9.95
	Reverse	19.73	0.79	66.99	10.53
10	Forward	19.82	0.75	60.78	8.99
	Reverse	19.58	0.75	54.58	8.05
15	Forward	19.61	0.68	45.29	6.04
	Reverse	19.48	0.71	44.68	6.18
20	Forward	19.10	0.66	40.96	5.14
	Reverse	19.09	0.65	40.86	5.05

Table S2. Photovoltaic parameters of PSCs fabricated on the double HTL ta-C (5, 10 nm) and NiO measured with forward and reverse scan directions.

Thickness [nm]		J_{SC} [mA/cm^2]	V_{OC} [V]	FF [%]	PCE [%]
5	Forward	22.51	1.01	77.28	17.52
	Reverse	22.86	1.01	74.65	17.21
10	Forward	19.60	1.00	73.22	14.30
	Reverse	18.97	1.01	73.13	14.05

Information Classification: General
